# How the temperate world was colonised by bindweeds: biogeography of the Convolvuleae (Convolvulaceae)

**DOI:** 10.1186/s12862-016-0591-6

**Published:** 2016-01-19

**Authors:** Thomas C. Mitchell, Bethany R. M. Williams, John R. I. Wood, David. J. Harris, Robert W. Scotland, Mark A. Carine

**Affiliations:** Plant Biodiversity Research, Technische Universität München, Emil-Ramann Strasse 2, 85354 Freising, Germany; Department of Plant Sciences, University of Oxford, South Parks Road, Oxford, OX1 3RB UK; Royal Botanic Garden Edinburgh, 20A Inverleith Row, Edinburgh, EH3 5LR UK; Department of Life Sciences, The Natural History Museum, Cromwell Road, London, SW7 5BD UK

**Keywords:** Amphitropical, *Calystegia*, *Convolvulus*, Disjunction, Diversification rates, *Polymeria*, Temperate, Transoceanic

## Abstract

**Background:**

At a global scale, the temperate zone is highly fragmented both between and within hemispheres. This paper aims to investigate how the world’s disjunct temperate zones have been colonised by the pan-temperate plant group Convolvuleae, sampling 148 of the *c*. 225 known species. We specifically determine the number and timing of amphitropical and transoceanic disjunctions, investigate the extent to which disjunctions in Convolvuleae are spatio-temporally congruent with those in other temperate plant groups and determine the impact of long-distance dispersal events on diversification rates.

**Results:**

Eight major disjunctions are observed in Convolvuleae: two Northern Hemisphere, two Southern Hemisphere and four amphitropical. Diversity in the Southern Hemisphere is largely the result of a single colonisation of Africa 3.1–6.4 Ma, and subsequent dispersals from Africa to both Australasia and South America. Speciation rates within this monophyletic, largely Southern Hemisphere group (1.38 species Myr^−1^) are found to be over twice those of the tribe as a whole (0.64 species Myr^-1)^. Increased speciation rates are also observed in *Calystegia* (1.65 species Myr^−1^).

**Conclusions:**

The Convolvuleae has colonised every continent of the world with a temperate biome in *c*. 18 Myr and eight major range disjunctions underlie this broad distribution. In keeping with other temperate lineages exhibiting disjunct distributions, long-distance dispersal is inferred as the main process explaining the patterns observed although for one American-Eurasian disjunction we cannot exclude vicariance. The colonisation of the temperate zones of the three southern continents within the last *c*. 4 Myr is likely to have stimulated high rates of diversification recovered in this group, with lineage accumulation rates comparable to those reported for adaptive radiations.

**Electronic supplementary material:**

The online version of this article (doi:10.1186/s12862-016-0591-6) contains supplementary material, which is available to authorized users.

## Background

The successful colonisation of temperate biomes by tropical lineages has involved the crossing of a significant physiological barrier that has acted as an important filter [1]. As a consequence, approximately half of all plant families remain restricted to the tropics [2]. Lineages that have made the transition to temperate biomes have experienced different fates with some lineages expanding their ranges to occupy highly disjunct areas where a suitable climate occurs, with distributions spanning both different continents and different hemispheres. Whilst the processes responsible for such patterns are complex [3–6], long-distance dispersal (LDD) events have been proposed for many such disjunctions and they may have acted as triggers for diversification [7, 8].

Thorne [9] recognised fifteen temperate disjunction patterns, several of which have since been the focus of molecular phylogenetic studies to understand the extent to which vicariance and dispersal explain biogeographic patterns in temperate plant lineages (e.g., the eastern North American–East Asian disjunction [10]; the temperate North and South American disjunction [11]; the western North American–East Asian disjunction [12]). One of the patterns documented by Thorne [9] was the ‘North–South Temperate disjunction’ which describes groups that are widespread in the northern temperate region and that also occur in one or more of the southern temperate zones (i.e., those located in South America, Africa and Australasia).

The Convolvuleae (Choisy) Choisy is one of 12 tribes within the predominantly tropical plant family Convolvulaceae Juss. [13]. It is one of only two tribes within the family to have widely colonised temperate regions (the other being the parasitic Cuscuteae) and it exhibits Thorne’s ‘North–south Temperate disjunction’ pattern. The Convolvuleae comprises three genera namely *Convolvulus* L., *Calystegia* R.Br. and *Polymeria* R.Br.. *Convolvulus* is the largest, comprising 190 species [14]. It has a main centre of diversity in the Mediterranean and western Asia, with further centres of diversity in eastern Asia and in temperate South America, southern and eastern Africa and Australasia; i.e., the three temperate zones of the southern hemisphere. Species also occur in North America, although they are few in number. *Calystegia* is readily distinguished from *Convolvulus* based on morphological characters (namely polypantoporate pollen and stigma shape) but molecular analyses suggest it is nested within the larger *Convolvulus* clade [13, 15, 16]. *Calystegia* is taxonomically complex [17] with *c.* 26 species and more than 65 distinct taxa currently accepted [18]. The centre of diversity for *Calystegia* is in California where nearly half of the described taxa occur [19]. Other centres of diversity for *Calystegia* are found in eastern Asia and, to a lesser extent Europe and the Mediterranean. *Calystegia* also occurs in temperate regions of the Southern Hemisphere. Finally, the Australasian endemic *Polymeria* is the smallest of the three genera of Convolvuleae with eight species recognised [18]. Molecular analyses place it as sister group to the remainder of Convolvuleae [13, 15, 16].

A recent study by Williams et al. [16] established a robust phylogenetic hypothesis of the Convolvuleae that sampled 62 % of species diversity in the tribe and was based on data from both the nuclear ITS region and the chloroplast *mat*K and *rbc*L regions. The goal of this paper is to utilise that phylogenetic framework to determine how the North–South Temperate disjunction pattern displayed by Convolvuleae was generated. Specifically, we aim to (i) determine the number, timing and cause (dispersal versus vicariance) of amphitropical and transoceanic disjunctions in the pan-temperate Convolvuleae and (ii) determine how major disjunctions in the history of the group may have impacted on diversification rates.

## Results

### Convolvulaceae–solanaceae analysis

An alignment comprising 153 species of Convolvulaceae (of which 109 were Convolvuleae; eight *Polymeria*, 11 *Calystegia*, 90 *Convolvulus*) and 343 species of Solanaceae (126 Solanoideae) and 1328 characters from the *mat*K and *rbc*L regions (of which 538 were parsimony informative) was used to establish divergence times within Convolvulaceae. The *rbc*L region was coded with missing data for 241 taxa, of which four were Convolvuleae. A chronogram with major groups is summarised in Additional file 1. The Convolvulaceae are resolved to have arisen 44.1 (95 % HPD 33.9–51.2) Ma, in agreement with Särkinen et al. [20]. Age estimates established for nodes within Solanaceae are also in agreement with Särkinen et al. [20]. Within Convolvulaceae, the Convolvuloideae *sensu* Stefanović et al. [21] is resolved as 20.9 (14.3–27.5) Myr old, with the Convolvuleae crown group (corresponding to the split between *Polymeria* and the *Convolvulus* + *Calystegia* clade) resolved at 17.9 (11.8–23.7) Ma.

### Convolvuleae analysis

**Fig. 1:**
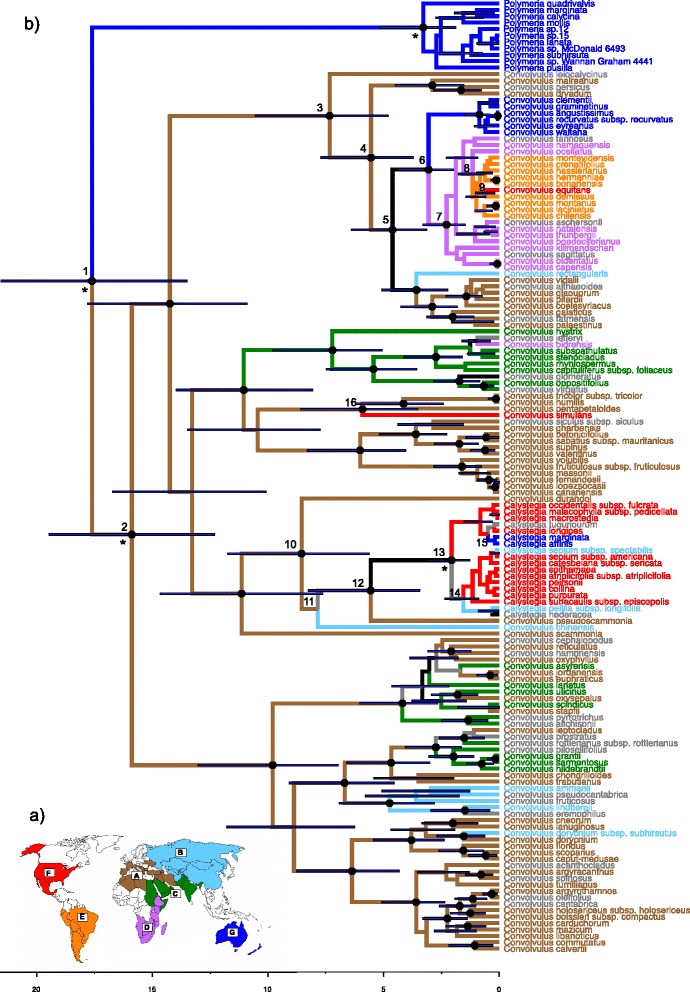
Phylogeny of Convolvuleae. **a**) Map depicting the areas of endemism for Convolvuleae delimited using UPGMA analysis. **b**) Dated phylogeny of Convolvuleae inferred in beast from analysis of the concatenated ITS, *mat*K and *rbc*L dataset. Node bars represent 95 % HPD estimates. Scale bar represent millions of years before present. Coloured branches and taxon names indicate the distribution area inferred in Lagrange, as shown in Fig. 1a. Black branches indicate ambiguous areas (less than 0.2 lnL difference between first and second most likely distribution). Grey branches indicate a multiple area distribution. Numbers at the top-left of nodes are referred to in Table 1. * indicate the location of calibrated nodes. Black circles on nodes indicate nodes with Bayesian Posterior Probabilities of at least 0.95

Five areas of endemism within Convolvuleae were delimited using UPGMA clustering of species by country distribution data (Fig. 1a). A Convolvuleae alignment consisted of 148 species of Convolvuleae (11 *Polymeria*, 18 *Calystegia,* 119 *Convolvulus*) and 2033 characters from the *rbc*L, *mat*K and ITS regions (matrix deposited in TreeBASE, study 18623). Divergence times estimated in beast using calibration points derived from the Solanaceae-Convolvulaceae analysis above and ancestral area reconstructions estimated using lagrange are provided in Fig. 1b with Table 1 summarising the information for key nodes of interest.

**Table 1:** Biogeographical inference and minimum age estimates for key nodes

	Lagrange analysis			Molecular dating using beast			
Node	Split	lnL	Rel. Prob.	Node BPP	Node age (Myr)	95 % HPD (Myr)	Notes
1	G/A	−219.5	0.5948	1	17.61	13.50–21.56	Convolvuleae crown group. Disjunction (i).
	G/AC	−221.3	0.1036				
2	A/A	−219.6	0.565	1	15.89	12.32–19.46	*Convolvulus* + *Calystegia* crown group.
3	A/A	−220.3	0.2739	1	7.34	4.8–10.55	
	A/ADG	−221.5	0.08379				
	ABC/A	−221.6	0.07558				
	A/AD	−222.1	0.04663				
	AC/A	−222.1	0.04593				
	AB/A	−222.1	0.04531				
	A/ABD	−222.2	0.0.04				
4	A/A	−220.3	0.2915	1	5.55	3.72–7.74	
	A/ADG	−220.9	0.1506				
	A/AD	−221.0	0.1379				
	A/ABD	−221.0	0.132				
	A/ABG	−221.7	0.06887				
	A/AG	−221.8	0.06279				
	A/AB	−221.8	0.06069				
5	D/AB	−220.3	0.2897	1	4.62	3.12–6.41	Stem of Southern Hemisphere group. Disjunction (ii).
	DG/A	−220.3	0.2887				
	D/A	−220.7	0.1846				
	G/AB	−221.1	0.1196				
	G/A	−222.1	0.04466				
6	G/D	−219.1	0.9433	1	3.06	1.99–4.41	Crown group of the circum-South Temperate (cST group), Southern-and-Eastern-African to Australasia. Disjunction (iii).
7	D/D	−219.1	0.9326	1	2.28	1.46–3.31	Southern-and-Eastern-African and South American crown group.
8	D/E	−219.1	0.9557	0.52	1.15	0.65–1.78	Southern-and-Eastern-African to South American movement. Disjunction (iv).
9	F/E	−219	1.0	0.59	0.57	0.18–1.03	South America to North America movement. Disjunction (v).
10	A/A	−219.8	0.4384	1	8.55	5.62–11.74	*Calystegia* and allies crown group.
	A/AB	−220.2	0.297				
	A/B	−220.8	0.1656				
11	AB/B	−219.5	0.5995	0.47	7.85		
	B/B	−220.9	0.1513				
12	BF/A	−219.9	0.4336	1	5.56	3.45–8.26	Central-and-North-Eastern-Asia to North America movement, *Calystegia* stem. Disjunction (vi).
	B/A	−219.9	0.3958				
13	F/BF	−219.5	0.6076	1	2.06	1.28–3.06	*Calystegia* crown group.
	F/BCF	−220.3	0.2656				
14	F/B	−219.9	0.4225	0.66	1.55	0.88–2.36	
	F/BC	−220.2	0.3018				
	BF/B	−220.6	0.2156				
15	EFG/G	−219.5	0.5969	0.79	0.82	0.3–1.53	Stem node of clade with Amphitropical American to Southern Hemisphere movement (South America and Australasia). Disjunction (vii).
	FG/G	−220.0	0.3934				
16	A/F	−219	1.0	1	5.92	3.53–8.6	Mediterranean-and-Middle-East to North America disjunction. Disjunction (viii).

Lagrange optimisations and beast minimum age estimates for key nodes in the Convolvuleae analysis. Node numbers are labelled in Fig. 1b. Lagrange splits refer to areas shown in Fig. 1a in the format x/y where x relates to the top branch, and y relates to the bottom branch exiting the labelled node. Log likelihoods (lnL) and relative probabilities (Rel. Prob.) are given for each Lagrange optimisation within two lnL of the most likely split optimisation. Bayesian Posterior Probabilities (BPP), mean node ages and 95 % highest posterior density (HPD) estimates inferred in beast are given for each node

The ancestral area of the *Convolvulus* + *Calystegia* clade is inferred to be the Mediterranean-and-Middle-East (area A in Fig. 1a; node 2). Dispersal between contiguous areas (i.e., A–B, A–C, C–D (Fig. 1a)) occurred frequently (Fig. 1b). Movement between disjunct (i.e., non-contiguous) areas has been much less common and eight such events are inferred. These are, in order of recency: (i) amphitropical disjunction between Australasia (Area G) and the Mediterranean-and-Middle-East (Area A) (posterior probability for node (PP) = 1) dated 17.61 Ma (95 % highest posterior density (HPD): 13.50–21.56 Ma) (node 1; Fig. 1b; Table 1), (ii) Northern hemisphere disjunction between the Mediterranean-and-Middle-East (A) and North America (F) (PP = 1) dated 5.92 Ma (3.53–8.6 Ma) (node 16), (iii) Northern hemisphere disjunction between Central-and-North-Eastern-Asia (B) and North America (F) (PP = 1), dated 5.56 Ma (3.45–8.26 Ma) (node 12), (iv) amphitropical disjunction between the Mediterranean-and-Middle-East (A) and Southern-and-Eastern-Africa (D) (PP = 1) dated 4.62 Ma (3.12–6.41 Ma) (node 5), (v) transoceanic southern hemisphere disjunction between Southern-and-Eastern-Africa (D) and Australasia (G) (PP = 1), dated 3.06 Ma (1.99–4.41 Ma) (node 6), (vi) weakly supported transoceanic southern hemisphere disjunction between Southern-and-Eastern-Africa (D) and South America (E) (PP = 0.52) dated as 1.15 Ma (0.65–1.78 Ma) (node 8), (vii) weakly supported (PP = 0.59) amphitropical disjunction between South America (E) and North America (F), dated 0.57 Ma (0.18–1.03 Ma) (node 9) and (viii) weakly supported (PP = 0.79) amphitropical disjunction between North America (F) and the Southern Hemisphere (Australasia (G), South America (E) or both, node 15), dated 0.82 Ma (0.3–1.53 Ma)

### Diversification rate analysis

**Fig. 2:**
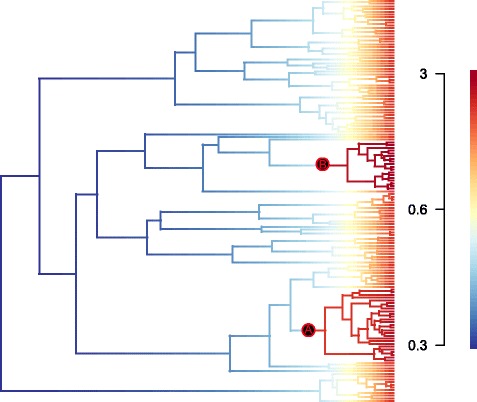
The maximum a posteriori probability rate shift configuration inferred by BAMM. The maximum a posteriori probability rate shift configuration inferred by BAMM analysis of the Convolvuleae concatenated ITS, *mat*K and *rbc*L dataset. Branches are coloured according to the rate inferred along that branch. Speciation rates are given as species Myr^−1^. Two rate shifts are inferred: **a**) the stem branch of the circum-South Temperate clade (Bayes factor 755); b**)** the stem branch of *Calystegia* (Bayes factor 424)

BAMM analysis found support for rate shifts within the Convolvuleae phylogeny compared with a single rate model (Bayes factors (BF) > 30 for 2–6 shifts). The maximum a posteriori (MAP) probability rate shift configuration, which alone explains 56 % of the data, infers two rate shifts: one on the stem branch of the southern hemisphere clade (Fig. 2; group A; BF = 755) and one on the stem branch of *Calystegia* (Fig. 2; group B; BF = 424) (Additional file 2a). Mean speciation rates (λ) within the circum-South Temperate (cST) clade (1.38, 90 % HPD: 0.71–2.07) and *Calystegia* (1.65, 90 % HPD: 0.61–2.73) are over twice those of the tribe as a whole (0.64, 90 % HPD: 0.5–0.83). Extinction rates (μ) however are also slightly higher in both the cST clade (0.47, 90 % HPD: 0.04–1.19) and *Calystegia* (0.86, 90 % HPD: 0.14–2.07) than in Convolvuleae as a whole (0.31, 90 % HPD: 0.11–0.58). Mean diversification rates (λ - μ) in the cST clade (0.91 species Myr^−1^) are therefore nearly three times greater than in the tree as a whole (0.34 species Myr^−1^), while those in *Calystegia* are over twice as fast (0.8 species Myr^−1^) as Convolvuleae in general. Extinction rates are inferred to have remained fairly constant over the history of the tribe, however speciation rates appear to have increased considerably in the last 2.5 Myr (Additional file 2b).

## Discussion

Both the world’s oceans and the equatorial tropics present barriers to dispersal of temperate lineages, potentially limiting exchange between the disjunct temperate zones of the world. In Convolvuleae, we observe four amphitropical and four transoceanic disjunctions in the history of the group with an increase in diversification rate associated with one amphitropical disjunction (the main group of *Convolvulus* in the southern hemisphere). A second increase in diversification rate is observed in *Calystegia* which also exhibits a transoceanic disjunction.

The four amphitropical disjunctions are spread throughout the history of the tribe (Fig. 1b, nodes 1, 5, 9 and 16). The earliest dates to the mid-Miocene (17.61 (13.5–21.56) Ma; node 1; Fig. 1b; Table 1) and separates the Australasian endemic *Polymeria* from the remainder of Convolvuleae (*Convolvulus* + *Calystegia*), for which the Mediterranean-and-Middle-East is resolved as the ancestral distribution area. This spatio-temporal pattern is consistent with the inferred timing of the disjunction between Australia and Eurasia in *Carex* subsect. *Spirostachyae* (*c*. 16–26 Ma [22]) and in *Halosarcia* (*c*. 15–20 Ma [23]). Escudero et al. [22] invoked LDD to explain this disjunction although the timing is coincident with the mid-Miocene Climatic Optimum 15–17 Ma, which saw the expansion of tropical forests, an event that is thought to have facilitated the dispersal of tropical plant and animal groups between Africa and Asia [24]. It is plausible that a corresponding contraction of temperate areas may have led to the disjunction apparent in these groups. Särkinen et al. [20] resolved a similar sister group relationship in *Solanum* between the Western Mediterranean–Macaronesian endemic Normania clade and the Australasian endemic Archaesolanum clade and an Australian–Northern Hemisphere disjunction was also inferred for *Atriplex* [25]. However, the timing of these was more recent (8.3 Ma and 9.8–7.8 Ma respectively) suggesting that the history of Australasian–Northern Hemisphere disjunctions is complex with multiple, temporally distinct events likely involved.

A second amphitropical disjunction in Convolvuleae is the result of the colonisation of the Southern Hemisphere by *Convolvulus* during the late Miocene to Pliocene *c*. 4.62 (3.12–6.41) Ma (node 5; Fig. 1b; Table 1). The most probable scenario involves dispersal from the Mediterranean-and-Middle-East into Southern-and-Eastern-Africa, followed by dispersal from there to Australasia *c*. 3.06 (1.99–4.41) Ma (node 6) and South America *c*. 1.15 (0.65–1.78) Ma (node 8) although the precise relationships of African and American taxa are not well supported. The mountains of the East African rift system, which link Southern Africa with the Horn of Africa are thought to have originated *c*. 12–40 Ma [26] and they provide a plausible trans-African dispersal corridor for Convolvuleae as has been suggested for other temperate taxa (e.g., *Senecio* [27]; *Disa*, Irideaeae p.p., *Pentaschistis*, Restionaceae [28]; *Androcymbium* [29]; *Scabiosa* [30]). It is notable that whilst some African amphitropical disjunctions are inferred to be pre-Pliocene (e.g., *Juniperus,* 30.5 (14.0–47.0) Ma [31]; *Hyacinthoideae*, 18.7 (18.8–18.7) Ma [32]; *Thamnosma*, 8.7 (5.3–12.1) Ma [33]), Plio– Pleistocene disjunctions, consistent with that observed in Convolvuleae have been reported in a number of groups. For example, colonisation of southern Africa from the north through this corridor has been inferred in *Apium* (4.1 (1.2–7.0) Ma [34]), *Ranunculus* (3.9 (2.6–5.3) Ma [35]) and *Scabiosa* (1.6 (0.7–2.6) Ma [30]) whilst South to North colonisation has been inferred for *Androcymbium* (4.0 (2.5–5.5) Ma and 3.0 (1.5–4.5) Ma [25]) and, very recently in *Senecio* (0.2 (0–0.4) Ma [27]).

The remaining amphitropical disjunctions are observed in the New World. Pleistocene dispersal (0.57 (0.18–1.03) Ma) from South America to North America is inferred in the cST group (node 9; Fig. 1b; Table 1). A southwards dispersal in *Calystegia* (node 15) is inferred to have occurred at a similar time *c*. 0.82 (0.3–1.53) Ma resulting in the colonisation of South America and Australasia. However, limited support (*Convolvulus*; PP = 0.59, *Calystegia*; PP = 0.79) or taxonomic uncertainty (*Calystegia*) means that these patterns should be interpreted with caution and the evolution and biogeography of the *Calystegia* clade in particular would benefit from further research.

These limitations notwithstanding, amphitropical American disjunctions of recent origin have been inferred in a range of groups with evidence for dispersal in both directions [4]. Bird mediated dispersal has frequently been proposed as responsible for such disjunctions due to the seasonal migration of birds between the Northern and Southern hemispheres (e.g., [11, 36, 37]). Whilst evidence for this is largely anecdotal, epizoochoric bird-mediated LDD between California and Chile has been demonstrated in *Lepidium* [36]. In the case of *Convolvulus,* viable seeds of *Convolvulus arvensis* have been recovered from the digestive tract of migratory killdeer (*Charadrius vociferus*) up to six days after ingestion [38]. Importantly however, long-distance internal transport of seeds, even in generally larger waterbirds has been shown to be limited to around 300 km making extreme long-distance endozoochoric dispersal unlikely [39]. Montane South American species of *Convolvulus* such as *C. crenatifolius* and *C. montanus* are frequently found above 1500 m [14] and the Andean high mountains, which are of late Miocene origin [40], may have provided a suitable route for the dispersal of temperate Convolvuleae lineages across the neotropics.

Remarkably few transoceanic dispersal events are necessary to explain the global distribution of the Convolvuleae, in contrast to groups such as Fabaeae [41]. Between the major Southern Hemisphere landmasses, we infer only two such dispersal events which both occurred 0.65–4.41 Ma, long after the breakup of the Gondwanan landmass and too recent to involve an Antarctic corridor [42]. Divergence time estimates for southern temperate plant groups indicate a wide range of ages, with Convolvuleae disjunctions among the most recent [42]. Whilst detailed information on dispersal mechanisms within the tribe are lacking, the variability of seed characteristics in Convolvulaceae [43] and evidence of long-distance oceanic seed dispersal elsewhere in the family [44] anecdotally support an oceanic dispersal hypothesis for the Southern Hemisphere distribution of Convolvuleae.

Two independent events in the late Miocene/Pliocene are inferred to be responsible for the earliest colonisation of North America. The most likely scenario for the *Calystegia* clade is dispersal from the Mediterranean-and-Middle-East into Eastern Asia *c*. 7.85 Ma (node 11; Fig. 1b; Table 1) and from there into North America *c*. 5.56 (3.45–8.26) Ma (node 12). The East Asian–North American disjunction is one of the best studied disjunctions with numerous examples of movement between the two regions throughout the Cenozoic [10]. The continents of the Northern Hemisphere were connected until 5.4–5.5 Ma when the Bering Land Bridge joining North America and Eastern Asia was finally severed [45]. A circum-Arctic floral region spanning this landmass prior to the severing of the land connections is frequently hypothesised as responsible for both the similarity and diversity of the flora in these regions (e.g., [9, 31, 46–48]). Given our estimated age for the Northern Hemisphere disjunction in Convolvuleae we are unable to reject a vicariance hypothesis for the origin of *Calystegia* in North America, in contrast to all other disjunctions we have inferred.

In the case of *Convolvulus simulans* (node 16), dispersal directly from the Mediterranean-and-Middle-East to North America *c*. 5.92 (3.53–8.6) Ma is inferred. A disjunction between the Mediterranean regions of North America and Europe (Madrean–Tethyan) is well documented (see [5]) and long distance dispersal to North America from the Mediterranean during the late Miocene/Pliocene has been inferred in a number of lineages (e.g., *Exaculum*/*Schenkia*–*Zeltnera c*. 9 Ma [49], *Crocanthemum–Hudsonia c*. 5.2–9.2 Ma [50], *Eobassia/Chenolea/Spirobassia–Neokochia c*. 8.8–13.1 Ma [51]).

With regards widespread and naturalised *Convolvulus* that were excluded from our analyses, comparison between the phylogeny estimated in Williams et al. [16] and our biogeographic inference suggests that both excluded species (*C. arvensis* and *C. lineatus*) probably originated in the Mediterranean-and-Middle-East region.

The diversification rate analysis reveals mean diversification rates for Convolvuleae of 0.34 species Myr^−1^, above the estimated diversification rates of angiosperms as a whole (0.077–0.089 species Myr^−1^, [52]. Furthermore, two shifts to increased diversification rates are supported within the tribe, with strong support for a rate shift in the southern hemisphere clade (Fig. 2; group A) leading to mean diversification rates (0.91 species Myr^−1^) which exceed those of adaptive radiations such as the Hawaiian radiation of *Bidens* (0.3–0.8 species Myr^−1^) [7]. BAMM suggests the elevated diversification rates are linked to an increase in speciation rate as opposed to a decrease in extinction rate (Table 2). The southern hemisphere clade contains at least two long-distance oceanic dispersal events within the Southern Hemisphere (nodes 6 and 8; Fig. 1b; Table 1) and at least two amphitropical dispersal events (nodes 5 and 9), with the shift to elevated diversification rates associated with the initial dispersal into the southern hemisphere in the late Miocene or Pliocene. This is consistent with other studies demonstrating the impact of Miocene dispersal events important in promoting diversification [8, 53].

**Table 2:** Convolvuleae diversification rates

Parameter		Convolvuleae	Clade A	Clade B
λ	mean	0.6415216	1.383068	1.658275
	5 %	0.5002688	0.7070913	0.6142909
	95 %	0.8320921	2.0741443	2.7378688
μ	mean	0.3056936	0.4715051	0.8596539
	5 %	0.1067923	0.0443146	0.1363267
	95 %	0.5751786	1.1944557	2.0701412
Mean net diversification (λ-μ)		0.335828	0.9115629	0.7986211

Estimated mean 90 % HPD (Highest Posterior Density) speciation (λ), extinction (μ) and net diversification rates inferred in BAMM for Convolvuleae, and two clades with shifts to increased diversification rates (Fg. 2; A and B). Rates are given in species Myr^−1^

The second diversification rate shift is observed in *Calystegia.* BAMM finds support, albeit less strongly, for a shift to increased diversification rates on the stem branch of *Calystegia* (Fig. 2; group B), leading to mean diversification rates (0.8 species Myr^−1^), over twice as high as those found in Convolvuleae as a whole. Most of the diversity of *Calystegia* is in North America and specifically California [19] and dispersal from East Asia into North America, again in the Miocene/Pliocene could also have been an important trigger for diversification within the group.

## Conclusions

In summary, our results indicate that the Convolvuleae has successfully colonised every continent of the world with a temperate biome in *c*. 18 Myr. The tropics and major oceans have been significant dispersal barriers for the group with only eight major disjunctions underlying this broad ‘North–south temperate’ distribution pattern. In keeping with many other disjunct temperate lineages, long-distance dispersal is inferred as the main process explaining the patterns observed although for one American-Eurasian disjunction we cannot exclude vicariance resulting from the severing of the Bering Land Bridge. Even though dispersal is the primary process generating the patterns observed, spatio-temperal congruence is observed with other temperate disjunct groups suggesting a common explanation for the patterns observed. The Convolvuleae exhibits high diversification rates overall when compared to other angiosperm groups and the colonisation of the temperate zones of the three southern continents within the last *c*. 4 Myr is associated with an increase in diversification rate with lineage accumulation rates in the clade comparable to those reported for adaptive radiations. The *Calystegia* clade also exhibits a high diversification rate which probably reflects rapid diversification following colonisation of western North America. The Convolvuleae thus provide a striking example of the ability of temperate lineages to rapidly colonise highly disjunct areas worldwide and to diversify.

## Methods

### Divergence time estimation

The fossil record of Convolvulaceae is poor with none of the fossils assigned to the family (*Convolvulites orichitus* [54], *Tricolpites trioblatus* [55], *Calystegiapollis microechinatus* (in [56]) able to be accurately placed within a phylogeny. We therefore adopted a two-step calibration procedure. We first utilised a recent phylogenetic study of Solanaceae, the sister group to Convolvulaceae [20] which reviewed all 50 of the known fossils assigned to the family, as the basis for calibration points for divergence time estimation within Convolvulaceae based on chloroplast data. Second, node age estimates from the chloroplast phylogeny were used to calibrate a combined nuclear ITS and plastid *mat*K and *rbc*L phylogeny of Convolvuleae.

*mat*K and *rbc*L sequences for Convolvuleae from Williams et al. [16] were manually aligned with sequences of the same regions for taxa across the remainder of the Convolvulaceae and Solanaceae, which were retrieved from GenBank. Details of all accessions sampled are included in Additional file 3. The datasets were concatenated, with taxa lacking *mat*K sequences excluded and taxa lacking *rbc*L sequences coded with missing data for this region. Due to the lower levels of variation in the *rbc*L region [16] the missing data is unlikely to have any significant impact on the tree topology, as it will be overridden by the signal from the *mat*K region [57].

Following Särkinen et al. [20], we used two calibration points reflecting the youngest age estimates of the oldest assignable fossils to constrain (i) the stem node of Solanoideae with a lognormal offset of 23.0 Ma, mean of 0.01, and standard deviation (SD) of 1.0 and (ii) the Solanaceae stem node with a lognormal offset of 46.0 Ma, mean of 0.01, and SD of 1.0. A gamma distribution (shape 0.001, scale 1000) was used as a prior for the mean mutation rate. Bayesian time estimation with an uncorrelated lognormal relaxed clock model was implemented in beast v1.8 [58]. Two independent Markov Chain Monte Carlo (MCMC) runs of 200 million generations, sampling every 10,000 generations were conducted using a Speciation: Birth–Death process tree prior and the GTR + I + G model. A run sampling only from the prior probabilities was also performed to evaluate the performance of the priors. Mixing of the chains and convergence were assessed using Tracer v1.6 [59] as was confirmation of an effective sample size (ESS) in the post burn-in samples exceeding 200 for all estimated parameters. The output tree files were combined using LogCombiner v1.8 (part of the beast software package) discarding the first 10 % of trees of each run as burn in. TreeAnnotator v1.8 (part of the beast software package) was used to combine post burn-in trees from the two runs, calculate the maximum clade credibility tree and the mean 95 % higher posterior density (HPD) intervals of node ages. Final trees were edited in FigTree v 1.4.0 [60].

A second divergence time analysis was performed on a concatenated ITS, *mat*K and *rbc*L dataset modified from Williams et al. [16] since the ITS region included greater taxon sampling within Convolvuleae (see Additional file 3 for sampling details). Due to the separate modes of evolution, the manually aligned matrix was partitioned into nuclear and plastid regions and parameters estimated independently. Analysis in beast followed the protocol for the Convolvulaceae–Solanaceae analysis except that minimum age estimates from the aforementioned analysis were used to constrain the Convolvuleae root node and *Polymeria*, *Convolvulus* + *Calystegia* and *Calystegia* crown nodes with normally distributed prior at 17.89 Ma (SD = 3.0), 4.76 Ma (SD = 1.5), 15.18 Ma (SD = 2.5), and 2.7 Ma (SD = 0.8) respectively, with the distribution reflecting the 95 % HPD estimates, and MCMC runs were reduced to 20 million generations, sampling every 1000 generations.

### Biogeographic analysis

The extant distributions of all accepted taxa at a country level were collated, largely from Wood et al. [14]. Widespread taxa and those for which the natural distribution may have been obscured by frequent introductions/naturalisations (*Convolvulus arvensis*, *Convolvulus lineatus*, *Calystegia soldanella*, *Calystegia pulchra*, *Calystegia sepium* subsp. *sepium*, *Calystegia sepium* subsp. *roseata* and all *Calystegia silvatica* subspecies) were excluded as were countries with only a single taxon present. Areas of endemism were then delimited using Unweighted Pair-Group Method with Arithmetic Mean (UPGMA) clustering of a taxon × country distribution matrix using the Sørensen–Dice coefficient [61, 62] in DendroUPGMA [63]. Seven areas were delimited (Fig. 1a) and each taxon was coded as belonging to one or more of these regions. Given our use of country borders as opposed to ecological boundaries to delimit areas, we only considered range shifts between non-contiguous regions as disjunctions.

The historical biogeography of the Convolvuleae was reconstructed using the dispersal–extinction–cladogenesis (DEC) model implemented in Lagrange v 2.0.1 [64], with taxon distributions coded as above. Three time slices were incorporated into the DEC model reflecting the presence or absence of Northern Hemisphere land connections between the Old and New Worlds. Subsequently, Northern America was isolated from the rest of the Northern Hemisphere 0–5.5 Ma, a connection existed between North America and Asia via the Bering Land Bridge (BLB) 5.5–15 Ma, and a connection also existed between North America and Europe via the North Atlantic Land Bridge (NALB) 15–25 Ma. All possible area combinations were permitted throughout. Dispersal probabilities followed Mao et al. [65], however given the relatively young age of Convolvuleae, and the subsequent reduction in major continental movements we simplified the model from five to three dispersal probabilities: 1.0 for connected areas, 0.1 for widely disjunct areas and 0.5 for three combinations of narrowly disjunct areas (area A–area D, area B–area F and area E–area F, Fig. 1b).

### Diversification rate analysis

Bayesian Analysis of Macroevolutionary Mixtures (BAMM) v2.0 [66] was used to model the dynamics of speciation and extinction on the time-calibrated Convolvuleae phylogenetic tree. Incomplete and non-random taxon sampling was incorporated directly into the likelihood calculations by utilising the recent monograph of *Convolvulus* [14] to place missing taxa into their respective clades. Two independent BAMM metropolis-coupled MCMC (MCMCMC) runs, with three heated and one cold chain, were run for 10 million generations and sampled every 1000 generations. Convergence of BAMM runs was assessed by computing ESS of log-likelihoods and numbers of shifts using the CODA library for R: both parameters had effective sample sizes > 1000. The first 10 % of samples were discarded as burn-in. Post-run analysis and visualisation was performed using the R package BAMMtools v2.0 [67].

## Additional files

The data sets supporting the results of this article are available in the TreeBASE repository, study 18623; http://purl.org/phylo/treebase/phylows/study/TB2:S18623 and the following additional files. Additional file 1:
**Dated phylogeny of Convolvulaceae and Solanaceae inferred in** **beast** **from analysis of the concatenated** ***mat*** **K and rbcL dataset.** Node bars represent 95 % HPD estimates. Bayesian Posterior Probabilities (BPP) ≥ 0.95 are given by their respective nodes. Scale bar represents millions of years before present. * indicate the location of fossil-calibrated nodes (Särkinen et al. [20]). + indicate the location of nodes used to calibrate the Convolvuleae phylogeny. (PDF 87 kb) Additional file 2:
**BAMM outputs.** Phylorate plots (2a) and speciation rate through time curve for Convolvuleae (2b). Additional file 2a represents the distinct shift configurations that account for 95% of the probability of the data (f-values denote the posterior probability of each shift configuration). Branches are scale colour-coded to indicate rate variation from red (acceleration) to blue (deceleration). Circles indicate the location of core rate shifts and are similarly colour-coded, with circle size proportional to the marginal probability of a shift. Additional file 2b represents a speciation rate through time curve (red) for Convolvuleae. Blue shading represents the confidence on speciation rate at any point in time. (ZIP 474 kb) Additional file 3:
**List of accessions included in this study.** GenBank numbers are provided in the respective columns. A dash indicates that no sequence data was included for that region. (DOCX 26 kb)
