# Supplementary material for: How the temperate world was colonised by bindweeds: biogeography of the Convolvuleae (Convolvulaceae)
Source: BMC Evol Biol. 2016 Jan 19;16:16. doi: 10.1186/s12862-016-0591-6 (PMC4719731; doi:10.1186/s12862-016-0591-6)

Appendix S2. Dated phylogeny of Convolvulaceae and Solanaceae inferred in BEAST from analysis of the concatenated matK and rbcL dataset. Node bars represent 95 % HPD estimates. Bayesian Posterior Probabilities (BPP) ≥ 0.95 are given by their respective nodes. Scale bar represent millions of years before present. * indicate the location of fossil-calibrated nodes (Särkinen et al., 2013). + indicate the location of nodes used to calibrate the Convolvuleae phylogeny.

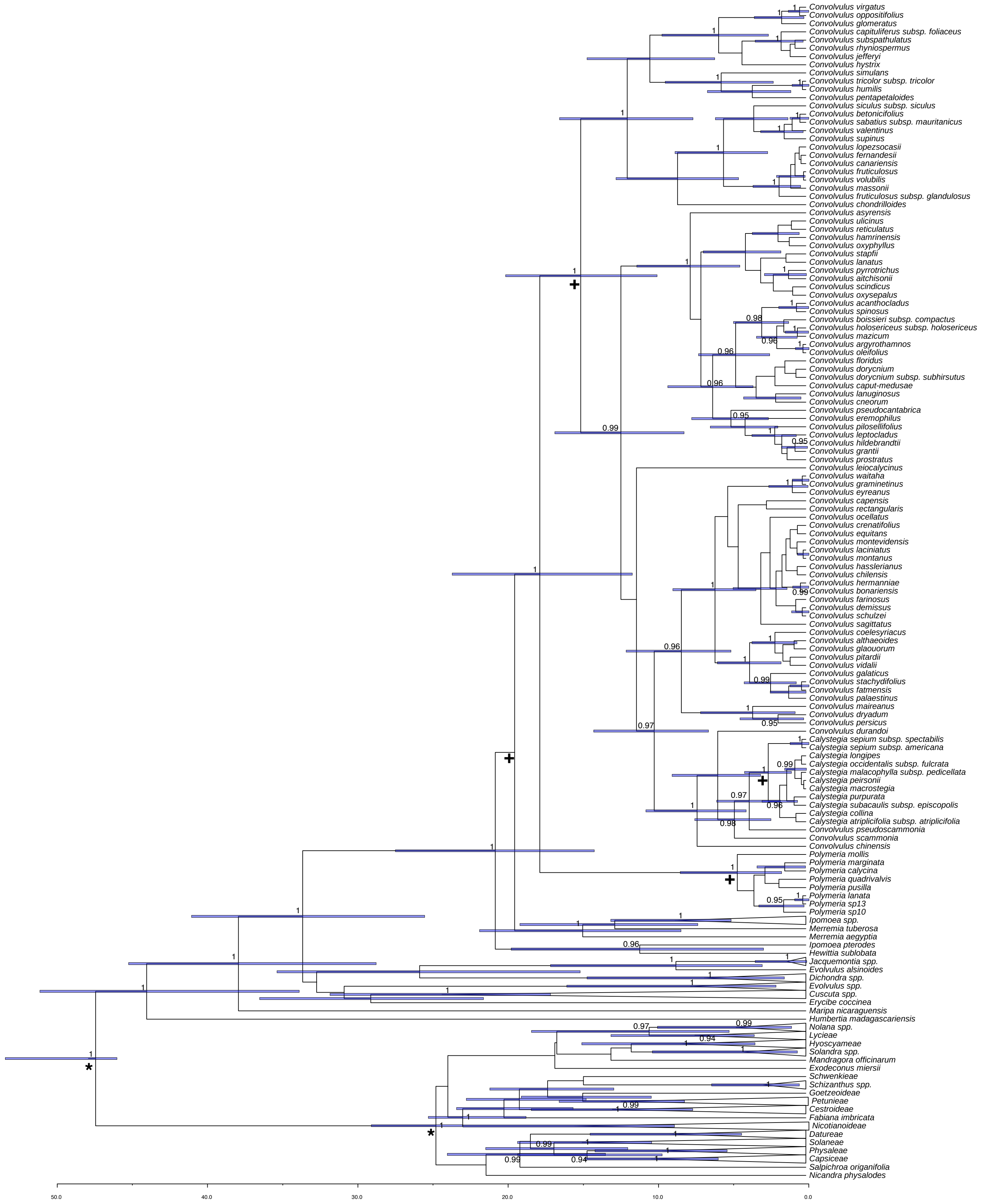

Supplement: Additional file 1: — Dated phylogeny of Convolvulaceae and Solanaceae inferred in beast from analysis of the concatenated mat K and rbcL dataset. Node bars represent 95 % HPD estimates. Bayesian Posterior Probabilities (BPP) ≥ 0.95 are given by their respective nodes. Scale bar represents millions of years before present. * indicate the location of fossil-calibrated nodes (Särkinen et al. [20]). + indicate the location of nodes used to calibrate the Convolvuleae phylogeny. (PDF 87 kb) [file 12862_2016_591_MOESM1_ESM.pdf]
