# Supplementary figures and images for: How the temperate world was colonised by bindweeds: biogeography of the Convolvuleae (Convolvulaceae)
Source: BMC Evol Biol. 2016 Jan 19;16:16. doi: 10.1186/s12862-016-0591-6 (PMC4719731; doi:10.1186/s12862-016-0591-6)

$f = 0.56$

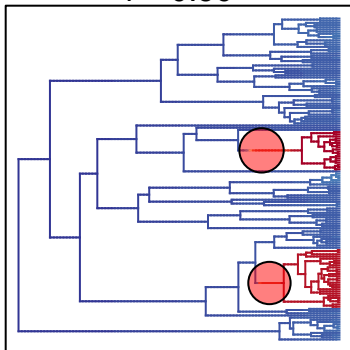

$f = 0.1$

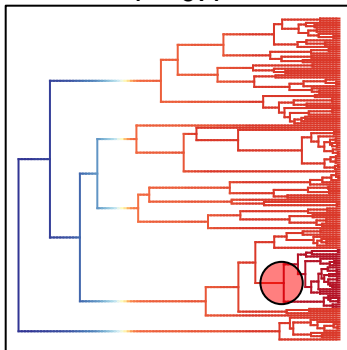

$f = 0.074$

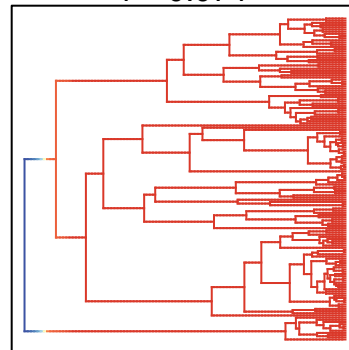

$f = 0.07$

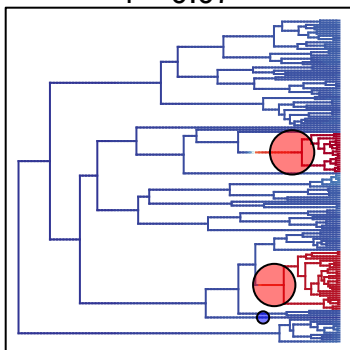

$f = 0.066$

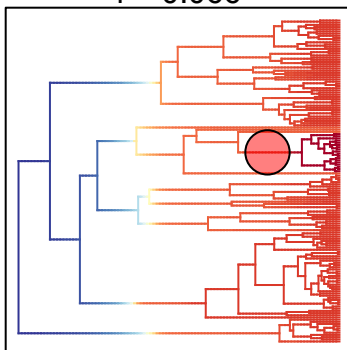

$f = 0.043$

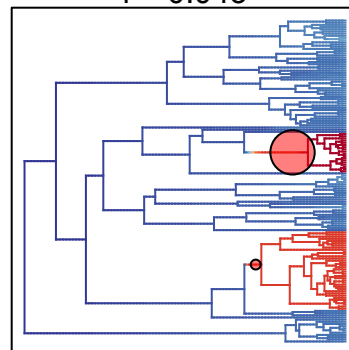

$f = 0.021$

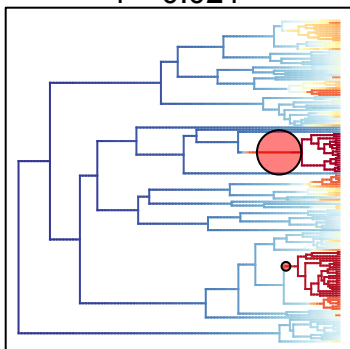

$f = 0.011$

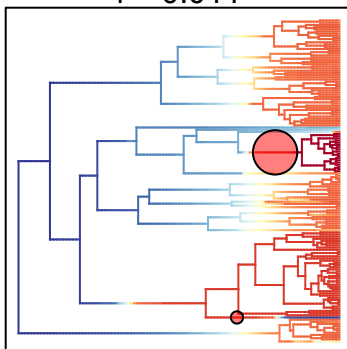

$f = 0.0094$

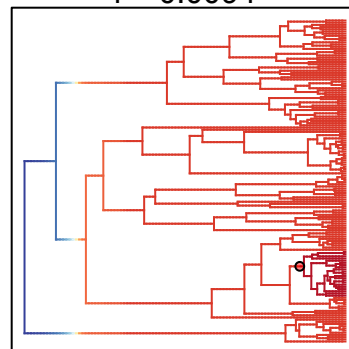

Supplement: Additional file 2: — BAMM outputs. Phylorate plots (2a) and speciation rate through time curve for Convolvuleae (2b). Additional file 2a represents the distinct shift configurations that account for 95% of the probability of the data (f-values denote the posterior probability of each shift configuration). Branches are scale colour-coded to indicate rate variation from red (acceleration) to blue (deceleration). Circles indicate the location of core rate shifts and are similarly colour-coded, with circle size proportional to the marginal probability of a shift. Additional file 2b represents a speciation rate through time curve (red) for Convolvuleae. Blue shading represents the confidence on speciation rate at any point in time. (ZIP 474 kb) [file 12862_2016_591_MOESM2_ESM.zip › 12862_2016_591_add3a.pdf]

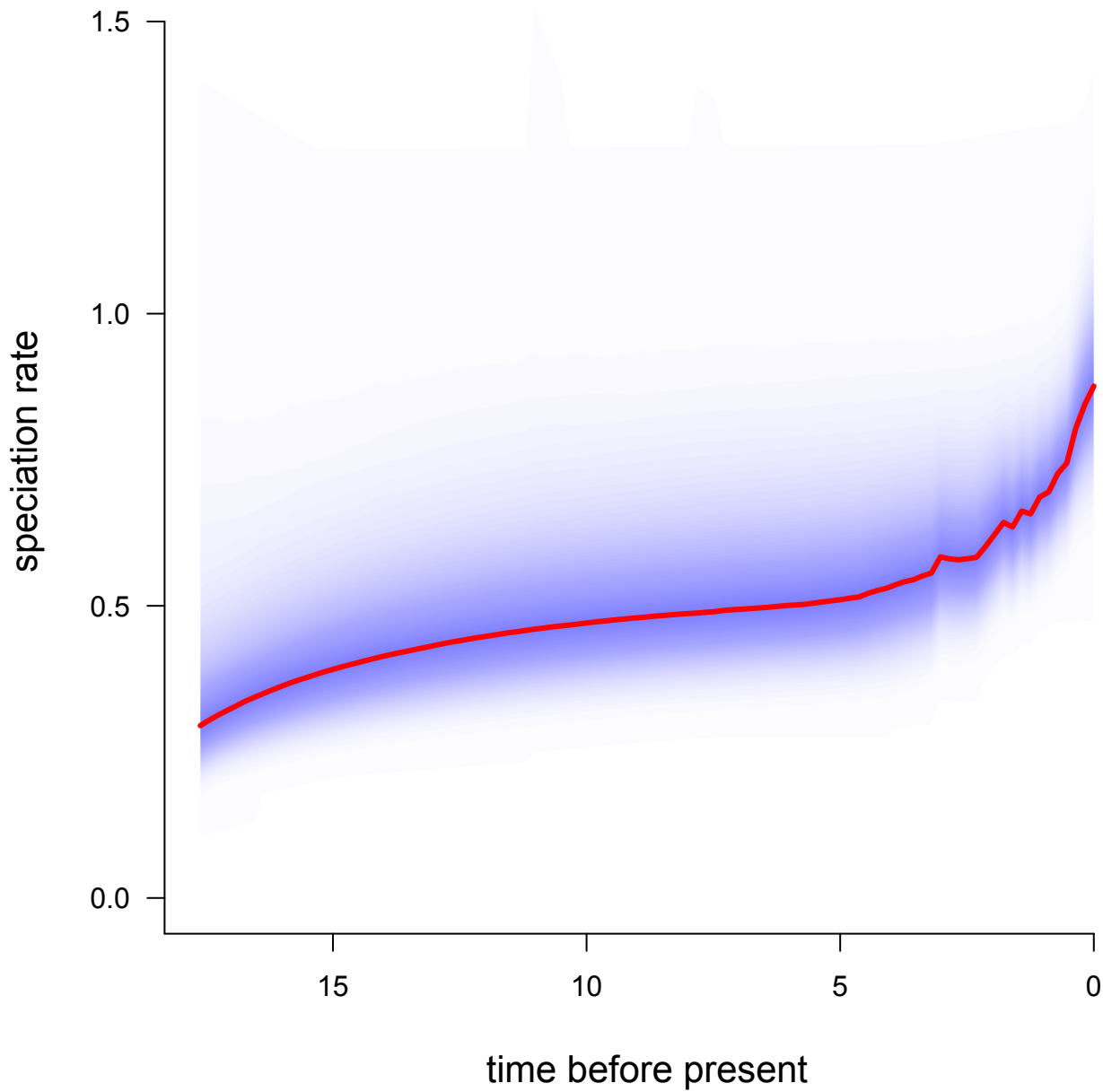

Supplement: Additional file 2: — BAMM outputs. Phylorate plots (2a) and speciation rate through time curve for Convolvuleae (2b). Additional file 2a represents the distinct shift configurations that account for 95% of the probability of the data (f-values denote the posterior probability of each shift configuration). Branches are scale colour-coded to indicate rate variation from red (acceleration) to blue (deceleration). Circles indicate the location of core rate shifts and are similarly colour-coded, with circle size proportional to the marginal probability of a shift. Additional file 2b represents a speciation rate through time curve (red) for Convolvuleae. Blue shading represents the confidence on speciation rate at any point in time. (ZIP 474 kb) [file 12862_2016_591_MOESM2_ESM.zip › 12862_2016_591_add3b.pdf]
