# Supplementary material for: How the temperate world was colonised by bindweeds: biogeography of the Convolvuleae (Convolvulaceae)
Source: BMC Evol Biol. 2016 Jan 19;16:16. doi: 10.1186/s12862-016-0591-6 (PMC4719731; doi:10.1186/s12862-016-0591-6)
Supplement: Additional file 3: — List of accessions included in this study. GenBank numbers are provided in the respective columns. A dash indicates that no sequence data was included for that region. (DOCX 26 kb) [file 12862_2016_591_MOESM3_ESM.docx]

**Additional file 3** List of accessions included in this study. GenBank numbers are provided in the respective columns. A dash indicates that no sequence data was included for that region.

| **Family** | **Species** | ***ITS*** | ***matK*** | ***rbcL*** |
| --- | --- | --- | --- | --- |
| Convolvulaceae | *Convolvulus acanthocladus* | KC528983 | KC529011 | KC529171 |
| Convolvulaceae | *Convolvulus aitchisonii* | KC528990 | KC529012 | KC529172 |
| Convolvulaceae | *Convolvulus althaeoides* | KC528819 | KC529013 | KC529173 |
| Convolvulaceae | *Convolvulus ammannii* | KC528945 | - | - |
| Convolvulaceae | *Convolvulus angustissimus* | KC528854 | - | - |
| Convolvulaceae | *Convolvulus argyracanthus* | KC528987 | - | - |
| Convolvulaceae | *Convolvulus argyrothamnos* | KC528972 | KC529017 | KC529177 |
| Convolvulaceae | *Convolvulus aschersonii* | KC528843 | - | - |
| Convolvulaceae | *Convolvulus asyrensis* | KC529000 | KC529021 | KC529181 |
| Convolvulaceae | *Convolvulus betonicifolius* | KC528904 | KC529022 | KC529182 |
| Convolvulaceae | *Convolvulus bidentatus* | KC528852 | - | - |
| Convolvulaceae | *Convolvulus bidrensis* | KC528872 | - | - |
| Convolvulaceae | *Convolvulus boedeckerianus* | KC528851 | - | - |
| Convolvulaceae | *Convolvulus boissieri subsp. compactus* | KC528977 | KC529035 | KC529194 |
| Convolvulaceae | *Convolvulus bonariensis* | KC528838 | KC529023 | KC529183 |
| Convolvulaceae | *Convolvulus calvertii* | KC528986 | - | - |
| Convolvulaceae | *Convolvulus canariensis* | KC528890 | KC529024 | KC529184 |
| Convolvulaceae | *Convolvulus cantabrica* | KC528968 | - | - |
| Convolvulaceae | *Convolvulus capensis* | KC528825 | KC529026 | KC529185 |
| Convolvulaceae | *Convolvulus capituliferus subsp. foliaceus* | KC528876 | KC529027 | KC529186 |
| Convolvulaceae | *Convolvulus caput-medusae* | KC528951 | KC529028 | KC529187 |
| Convolvulaceae | *Convolvulus carduchorum* | KC528981 | - | - |
| Convolvulaceae | *Convolvulus cephalopodus* | KC529004 | - | - |
| Convolvulaceae | *Convolvulus chilensis* | KC528831 | KC529029 | KC529188 |
| Convolvulaceae | *Convolvulus chinensis* | KC528928 | KC529031 | KC529190 |
| Convolvulaceae | *Convolvulus chondrilloides* | KC528942 | KC529032 | KC529191 |
| Convolvulaceae | *Convolvulus clementii* | KC528858 | - | - |
| Convolvulaceae | *Convolvulus cneorum* | KC528950 | KC529033 | KC529192 |
| Convolvulaceae | *Convolvulus coelesyriacus* | KC528823 | KC529034 | KC529193 |
| Convolvulaceae | *Convolvulus commutatus* | KC528984 | - | - |
| Convolvulaceae | *Convolvulus crenatifolius* | KC528827 | KC529038 | KC529197 |
| Convolvulaceae | *Convolvulus demissus* | KC528836 | KC529039 | KC529198 |
| Convolvulaceae | *Convolvulus dorycnium* | KC528957 | KC529040 | KC529199 |
| Convolvulaceae | *Convolvulus dorycnium subsp. subhirsutus* | KC528960 | KC529127 | KC529282 |
| Convolvulaceae | *Convolvulus dryadum* | KC528863 | KC529042 | KC529201 |
| Convolvulaceae | *Convolvulus durandoi* | KC528908 | KC529043 | KC529202 |
| Convolvulaceae | *Convolvulus equitans* | KC528832 | KC529044 | KC529203 |
| Convolvulaceae | *Convolvulus eremophilus* | KC528948 | KC529072 | KC529230 |
| Convolvulaceae | *Convolvulus euphraticus* | KC529008 | - | - |
| Convolvulaceae | *Convolvulus eyreanus* | KC528856 | KC529048 | KC529207 |
| Convolvulaceae | *Convolvulus farinosus* | KC528840 | KC529049 | KC529208 |
| Convolvulaceae | *Convolvulus fatmensis* | KC528862 | KC529052 | KC529211 |
| Convolvulaceae | *Convolvulus fernandesii* | KC528895 | KC529053 | KC529212 |
| Convolvulaceae | *Convolvulus floridus* | KC528955 | KC529055 | KC529214 |
| Convolvulaceae | *Convolvulus fruticosus* | KC528944 | KC529056 | KC529215 |
| Convolvulaceae | *Convolvulus fruticulosus subsp. fruticulosus* | KC528892 | KC529058 | KC529217 |
| Convolvulaceae | *Convolvulus galaticus* | KC528859 | KC529057 | KC529216 |
| Convolvulaceae | *Convolvulus gharbensis* | KC528900 | - | - |
| Convolvulaceae | *Convolvulus glaouorum* | - | KC529059 | KC529218 |
| Convolvulaceae | *Convolvulus glomeratus* | KC528886 | KC529060 | KC529219 |
| Convolvulaceae | *Convolvulus graminetinus* | KC528853 | KC529062 | KC529221 |
| Convolvulaceae | *Convolvulus grantii* | KC528935 | KC529063 | KC529222 |
| Convolvulaceae | *Convolvulus hamrinensis* | KC528995 | KC529064 | KC529223 |
| Convolvulaceae | *Convolvulus hasslerianus* | KC528834 | KC529065 | KC529224 |
| Convolvulaceae | *Convolvulus hermanniae* | KC528829 | KC529066 | KC529225 |
| Convolvulaceae | *Convolvulus hildebrandtii* | KC528937 | KC529054 | KC529213 |
| Convolvulaceae | *Convolvulus holosericeus subsp. holosericeus* | KC528974 | KC529067 | - |
| Convolvulaceae | *Convolvulus humilis* | KC528871 | KC529068 | KC529226 |
| Convolvulaceae | *Convolvulus hystrix* | KC528888 | KC529069 | KC529227 |
| Convolvulaceae | *Convolvulus jefferyi* | KC528875 | KC529071 | KC529229 |
| Convolvulaceae | *Convolvulus jordanensis* | KC529009 | - | - |
| Convolvulaceae | *Convolvulus kilimandschari* | KC528844 | - | - |
| Convolvulaceae | *Convolvulus laciniatus* | KC528830 | KC529074 | KC529232 |
| Convolvulaceae | *Convolvulus lanatus* | KC529002 | KC529075 | KC529233 |
| Convolvulaceae | *Convolvulus lanuginosus* | KC528959 | KC529076 | KC529234 |
| Convolvulaceae | *Convolvulus leiocalycinus* | KC528866 | KC529077 | KC529235 |
| Convolvulaceae | *Convolvulus leptocladus* | KC528941 | KC529078 | KC529236 |
| Convolvulaceae | *Convolvulus lopezsocasii* | - | KC529082 | KC529240 |
| Convolvulaceae | *Convolvulus libanoticus* | KC528980 | - | - |
| Convolvulaceae | *Convolvulus lindbergii* | KC528947 | - | - |
| Convolvulaceae | *Convolvulus lopezsocasii* | KC528894 | - | - |
| Convolvulaceae | *Convolvulus maireanus* | KC528865 | KC529083 | KC529241 |
| Convolvulaceae | *Convolvulus massonii* | KC528896 | KC529084 | KC529242 |
| Convolvulaceae | *Convolvulus mazicum* | KC528978 | KC529085 | KC529243 |
| Convolvulaceae | *Convolvulus montanus* | - | KC529086 | KC529244 |
| Convolvulaceae | *Convolvulus montevidensis* | KC528828 | KC529087 | - |
| Convolvulaceae | *Convolvulus namaquensis* | KC528842 | - | - |
| Convolvulaceae | *Convolvulus natalensis* | KC528850 | - | - |
| Convolvulaceae | *Convolvulus ocellatus* | - | KC529088 | KC529245 |
| Convolvulaceae | *Convolvulus oleifolius* | KC528970 | KC529089 | KC529246 |
| Convolvulaceae | *Convolvulus oppositifolius* | KC528879 | KC529090 | KC529247 |
| Convolvulaceae | *Convolvulus oxyphyllus* | KC528993 | KC529094 | KC529251 |
| Convolvulaceae | *Convolvulus oxysepalus* | KC529010 | KC529096 | KC529253 |
| Convolvulaceae | *Convolvulus palaestinus* | - | KC529097 | KC529254 |
| Convolvulaceae | *Convolvulus pentapetaloides* | KC528869 | KC529098 | KC529255 |
| Convolvulaceae | *Convolvulus persicus* | KC528864 | KC529100 | KC529257 |
| Convolvulaceae | *Convolvulus pilosellifolius* | KC528932 | KC529016 | KC529176 |
| Convolvulaceae | *Convolvulus pitardii* | KC528824 | KC529101 | KC529258 |
| Convolvulaceae | *Convolvulus prostratus* | KC528939 | KC529103 | KC529260 |
| Convolvulaceae | *Convolvulus pseudocantabrica* | KC528946 | KC529104 | KC529261 |
| Convolvulaceae | *Convolvulus pseudoscammonia* | KC528929 | KC529105 | KC529262 |
| Convolvulaceae | *Convolvulus pyrrotrichus* | KC528991 | KC529107 | KC529264 |
| Convolvulaceae | *Convolvulus rectangularis* | KC528868 | KC529108 | KC529265 |
| Convolvulaceae | *Convolvulus recurvatus subsp. recurvatus* | KC528855 | - | - |
| Convolvulaceae | *Convolvulus reticulatus* | KC528996 | KC529110 | KC529267 |
| Convolvulaceae | *Convolvulus rhyniospermus* | - | KC529112 | - |
| Convolvulaceae | *Convolvulus rottlerianus subsp. rottlerianus* | KC528940 | - | - |
| Convolvulaceae | *Convolvulus sabatius subsp. mauritanicus* | - | KC529114 | KC529270 |
| Convolvulaceae | *Convolvulus sagittatus* | KC528845 | KC529115 | KC529271 |
| Convolvulaceae | *Convolvulus sarmentosus* | KC528936 | - | - |
| Convolvulaceae | *Convolvulus scammonia* | KC528927 | KC529118 | KC529273 |
| Convolvulaceae | *Convolvulus schulzei* | - | KC529119 | KC529274 |
| Convolvulaceae | *Convolvulus scindicus* | KC529007 | KC529120 | KC529275 |
| Convolvulaceae | *Convolvulus scoparius* | KC528952 | - | - |
| Convolvulaceae | *Convolvulus siculus subsp. siculus* | KC528899 | KC529121 | KC529276 |
| Convolvulaceae | *Convolvulus simulans* | - | KC529122 | KC529277 |
| Convolvulaceae | *Convolvulus spinosus* | KC528988 | KC529123 | KC529278 |
| Convolvulaceae | *Convolvulus stachydifolius* | - | KC529124 | KC529279 |
| Convolvulaceae | *Convolvulus stapfii* | - | KC529125 | KC529280 |
| Convolvulaceae | *Convolvulus stenocladus* | KC528874 | - | - |
| Convolvulaceae | *Convolvulus subspathulatus* | KC528873 | KC529128 | KC529283 |
| Convolvulaceae | *Convolvulus supinus* | KC528901 | KC529129 | KC529284 |
| Convolvulaceae | *Convolvulus thunbergii* | KC528849 | - | - |
| Convolvulaceae | *Convolvulus trabutianus* | KC528943 | - | - |
| Convolvulaceae | *Convolvulus tricolor subsp. tricolor* | KC528870 | KC529131 | KC529286 |
| Convolvulaceae | *Convolvulus turrillianus* | KC528989 | - | - |
| Convolvulaceae | *Convolvulus ulicinus* | KC529006 | KC529132 | KC529287 |
| Convolvulaceae | *Convolvulus valentinus* | KC528902 | KC529133 | KC529288 |
| Convolvulaceae | *Convolvulus vidalii* | KC528820 | KC529134 | KC529289 |
| Convolvulaceae | *Convolvulus virgatus* | KC528883 | KC529135 | KC529290 |
| Convolvulaceae | *Convolvulus volubilis* | KC528898 | KC529137 | KC529292 |
| Convolvulaceae | *Convolvulus waitaha* | KC528857 | KC529138 | KC529293 |
| Convolvulaceae | *Calystegia affinis* | JF950722 | - | - |
| Convolvulaceae | *Calystegia atriplicifolia subsp. atriplicifolia* | KC528915 | KC529139 | KC529294 |
| Convolvulaceae | *Calystegia catesbeiana subsp. sericata* | KC528916 | - | - |
| Convolvulaceae | *Calystegia collina* | - | KC529140 | KC529295 |
| Convolvulaceae | *Calystegia hederacea* | JQ062467 | - | - |
| Convolvulaceae | *Calystegia longipes* | - | KC529141 | KC529296 |
| Convolvulaceae | *Calystegia macrostegia* | KC528923 | KC529145 | KC529300 |
| Convolvulaceae | *Calystegia malacophylla subsp. pedicellata* | KC528924 | KC529146 | KC529301 |
| Convolvulaceae | *Calystegia marginata* | EU812826 | - | - |
| Convolvulaceae | *Calystegia occidentalis subsp. fulcrata* | - | KC529147 | KC529302 |
| Convolvulaceae | *Calystegia peirsonii* | KC528914 | KC529148 | KC529303 |
| Convolvulaceae | *Calystegia pellita subsp. longifolia* | KC528920 | - | - |
| Convolvulaceae | *Calystegia purpurata* | - | KC529142 | KC529297 |
| Convolvulaceae | *Calystegia sepium subsp. americana* | KC528910 | KC529150 | KC529305 |
| Convolvulaceae | *Calystegia sepium subsp. spectabilis* | - | KC529156 | KC529311 |
| Convolvulaceae | *Calystegia spithamaea* | KC528913 | - | - |
| Convolvulaceae | *Calystegia subacaulis subsp. episcopolis* | KC528917 | KC529162 | KC529316 |
| Convolvulaceae | *Calystegia tuguriorum* | EU812835 | - | - |
| Convolvulaceae | *Polymeria calycina* | KC528813 | KC529163 | KC529317 |
| Convolvulaceae | *Polymeria lanata* | KC528814 | KC529164 | KC529318 |
| Convolvulaceae | *Polymeria marginata* | - | KC529165 | KC529319 |
| Convolvulaceae | *Polymeria mollis* | KC528816 | KC529166 | KC529320 |
| Convolvulaceae | *Polymeria pusilla* | - | KC529167 | KC529321 |
| Convolvulaceae | *Polymeria quadrivalvis* | - | KC529168 | - |
| Convolvulaceae | *Polymeria sp10* | - | KC529169 | KC529322 |
| Convolvulaceae | *Polymeria sp12* | KC528812 | - | - |
| Convolvulaceae | *Polymeria sp13* | - | KC529170 | KC529323 |
| Convolvulaceae | *Polymeria sp15* | KC528815 | - | - |
| Convolvulaceae | *Polymeria subhirsuta* | KC528811 | - | - |
| Solanaceae | *Anisodus luridus* | - | EF438901 | HQ216115 |
| Solanaceae | *Anthocercis angustifolia* | - | AJ585857 | - |
| Solanaceae | *Anthocercis gracilis* | - | AJ585858 | - |
| Solanaceae | *Anthocercis ilicifolia* | - | AJ585859 | - |
| Solanaceae | *Anthocercis intricata* | - | AJ585861 | - |
| Solanaceae | *Anthocercis littorea* | - | AJ585860 | - |
| Solanaceae | *Anthocercis myosotidea* | - | AJ585874 | - |
| Solanaceae | *Anthocercis sylvicola* | - | AJ585862 | - |
| Solanaceae | *Anthocercis viscosa* | - | AJ585863 | AVU08608 |
| Solanaceae | *Anthotroche blackii* | - | AJ585864 | - |
| Solanaceae | *Anthotroche myoporoides* | - | AJ585865 | - |
| Solanaceae | *Anthotroche pannosa* | - | AJ585866 | - |
| Solanaceae | *Anthotroche walcottii* | - | AJ585867 | - |
| Solanaceae | *Atropa belladonna* | - | AJ585882 | ABU08609 |
| Solanaceae | *Aureliana fasciculata var. fasciculata* | - | EF537319 | - |
| Solanaceae | *Browallia americana* | - | EF439050 | - |
| Solanaceae | *Brugmansia arborea* | - | GQ434219 | JN244363 |
| Solanaceae | *Brugmansia aurea* | - | EF438848 | - |
| Solanaceae | *Brugmansia suaveolens* | - | HM851090 | HM849829 |
| Solanaceae | *Brunfelsia portoricensis* | - | HM446659 | HM446761 |
| Solanaceae | *Calibrachoa parviflora* | - | EF438982 | - |
| Solanaceae | *Capsicum annuum* | - | AB721552 | JN114794 |
| Solanaceae | *Capsicum baccatum* | - | AB721620 | CBU08610 |
| Solanaceae | *Capsicum campylopodium* | - | EF537295 | - |
| Solanaceae | *Capsicum chacoense* | - | EF537279 | - |
| Solanaceae | *Capsicum chinense* | - | AB721662 | JX856309 |
| Solanaceae | *Capsicum eximium* | - | EF537297 | - |
| Solanaceae | *Capsicum flexuosum* | - | EF537280 | - |
| Solanaceae | *Capsicum frutescens* | - | AB721832 | JX996068 |
| Solanaceae | *Capsicum hunzikerianum* | - | EF537284 | - |
| Solanaceae | *Capsicum parvifolium* | - | EF537293 | - |
| Solanaceae | *Capsicum pereirae* | - | EF537303 | - |
| Solanaceae | *Capsicum pubescens* | - | AB721886 | AB721887 |
| Solanaceae | *Capsicum recurvatum* | - | EF537286 | - |
| Solanaceae | *Capsicum rhomboideum* | - | EF537289 | - |
| Solanaceae | *Capsicum schottianum* | - | EF537305 | - |
| Solanaceae | *Capsicum tovarii* | - | EF537268 | - |
| Solanaceae | *Capsicum villosum* | - | EF537304 | - |
| Solanaceae | *Cestrum diurnum* | - | GU135071 | - |
| Solanaceae | *Cestrum elegans* | - | AJ585891 | - |
| Solanaceae | *Cestrum fragile* | - | JQ589572 | - |
| Solanaceae | *Cestrum glanduliferum* | - | JQ589214 | - |
| Solanaceae | *Cestrum laevigatum* | - | JX517961 | - |
| Solanaceae | *Cestrum macrophyllum* | - | HM446667 | - |
| Solanaceae | *Cestrum parqui* | - | EF439054 | - |
| Solanaceae | *Crenidium spinescens* | - | AJ585868 | - |
| Convolvulaceae | *Cuscuta epilinum* | - | EU330281 | - |
| Convolvulaceae | *Cuscuta europaea* | - | EU330282 | - |
| Convolvulaceae | *Cuscuta japonica* | - | EU330283 | - |
| Convolvulaceae | *Cuscuta lupuliformis* | - | EU330284 | - |
| Convolvulaceae | *Cuscuta nitida* | - | EU330280 | - |
| Convolvulaceae | *Cuscuta reflexa* | - | EU330285 | - |
| Solanaceae | *Cyphanthera albicans* | - | AJ585870 | - |
| Solanaceae | *Cyphanthera anthocercidea* | - | AJ585869 | - |
| Solanaceae | *Cyphanthera microphylla* | - | AJ585873 | - |
| Solanaceae | *Cyphanthera odgersii* | - | AJ585875 | - |
| Solanaceae | *Datura arenicola* | - | KC146592 | - |
| Solanaceae | *Datura ceratocaula* | - | KC146593 | - |
| Solanaceae | *Datura discolor* | - | KC146594 | - |
| Solanaceae | *Datura ferox* | - | KC146595 | - |
| Solanaceae | *Datura inoxia* | - | JX996065 | - |
| Solanaceae | *Datura kymatocarpa* | - | KC146597 | - |
| Solanaceae | *Datura lanosa* | - | KC146598 | - |
| Solanaceae | *Datura metel* | - | GQ434221 | - |
| Solanaceae | *Datura pruinosa* | - | KC146600 | - |
| Solanaceae | *Datura quercifolia* | - | KC146601 | - |
| Solanaceae | *Datura reburra* | - | KC146602 | - |
| Solanaceae | *Datura stramonium* | - | HM851091 | - |
| Solanaceae | *Datura wrightii* | - | KC146604 | - |
| Convolvulaceae | *Dichondra carolinensis* | - | EU330287 | - |
| Convolvulaceae | *Dichondra micrantha* | - | HM850895 | HM849950 |
| Solanaceae | *Duboisia leichhardtii* | - | AJ585872 | - |
| Solanaceae | *Duboisia myoporoides* | - | AJ585871 | - |
| Solanaceae | *Dunalia fasciculata* | - | EF438836 | - |
| Convolvulaceae | *Erycibe coccinea* | - | HQ384568 | HQ384919 |
| Convolvulaceae | *Evolvulus alsinoides* | - | JQ587281 | JQ591144 |
| Convolvulaceae | *Evolvulus nummularius* | - | JQ587284 | JQ591145 |
| Convolvulaceae | *Evolvulus pilosus* | - | HQ384566 | HQ384917 |
| Solanaceae | *Exodeconus miersii* | - | EF438986 | - |
| Solanaceae | *Fabiana imbricata* | - | EF438938 | - |
| Solanaceae | *Goetzea elegans* | - | HQ384563 | AF035738 |
| Solanaceae | *Grammosolen dixonii* | - | AJ585876 | - |
| Solanaceae | *Grammosolen truncatus* | - | AJ585877 | - |
| Convolvulaceae | *Hewittia sublobata* | - | FJ795796 | - |
| Convolvulaceae | *Humbertia madagascariensis* | - | EU330288 | AY101062 |
| Solanaceae | *Hyoscyamus albus* | - | HM851096 | HQ216122 |
| Solanaceae | *Hyoscyamus aureus* | - | EF438841 | HQ216121 |
| Solanaceae | *Hyoscyamus niger* | - | EF438829 | HQ216125 |
| Solanaceae | *Iochroma australe* | - | EF438832 | - |
| Convolvulaceae | *Ipomoea alba* | - | JQ587689 | AY100963 |
| Convolvulaceae | *Ipomoea aquatica* | - | GU135084 | AY100958 |
| Convolvulaceae | *Ipomoea batatas* | - | AJ429355 | AY100962 |
| Convolvulaceae | *Ipomoea cairica* | - | FJ795793 | JN114807 |
| Convolvulaceae | *Ipomoea carnea subsp. fistulosa* | - | GU135080 | GU135243 |
| Convolvulaceae | *Ipomoea crepidiformis* | - | FJ795789 | - |
| Convolvulaceae | *Ipomoea dumosa* | - | JQ587292 | JQ591152 |
| Convolvulaceae | *Ipomoea hederifolia* | - | FJ795791 | - |
| Convolvulaceae | *Ipomoea hildebrandtii* | - | FJ795782 | - |
| Convolvulaceae | *Ipomoea imperati* | - | HM850896 | HM850072 |
| Convolvulaceae | *Ipomoea indica* | - | FJ795792 | HM850073 |
| Convolvulaceae | *Ipomoea lindenii* | - | JQ587295 | JQ591155 |
| Convolvulaceae | *Ipomoea mauritiana* | - | AY491651 | - |
| Convolvulaceae | *Ipomoea nil* | - | GQ434278 | GQ436722 |
| Convolvulaceae | *Ipomoea obscura* | - | FJ795784 | AY100968 |
| Convolvulaceae | *Ipomoea pterodes* | - | JQ587298 | JQ591157 |
| Convolvulaceae | *Ipomoea purpurea* | - | GU266612 | GQ436725 |
| Convolvulaceae | *Ipomoea repens* | - | FJ795795 | - |
| Convolvulaceae | *Ipomoea rubens* | - | FJ795788 | - |
| Convolvulaceae | *Ipomoea sinensis* | - | FJ795787 | - |
| Convolvulaceae | *Ipomoea spathulata* | - | FJ795781 | - |
| Convolvulaceae | *Ipomoea stenobasis* | - | FJ795790 | - |
| Convolvulaceae | *Ipomoea tenuirostris* | - | FJ795786 | - |
| Convolvulaceae | *Ipomoea trifida* | - | JQ587301 | JQ591160 |
| Convolvulaceae | *Jacquemontia reclinata* | - | HQ384567 | HQ384918 |
| Convolvulaceae | *Jacquemontia tamnifolia* | - | EU330286 | AY101037 |
| Solanaceae | *Jaltomata dentata* | - | EF438985 | - |
| Solanaceae | *Jaltomata hunzikeri* | - | EF438939 | - |
| Solanaceae | *Jaltomata procumbens* | - | KC146605 | - |
| Solanaceae | *Jaltomata repandidentata* | - | JQ589228 | JQ594124 |
| Convolvulaceae | *Lepistemon owariensis* | - | FJ795785 | AY100969 |
| Solanaceae | *Lycianthes biflora* | - | EF438838 | - |
| Solanaceae | *Lycianthes dejecta* | - | EF438843 | - |
| Solanaceae | *Lycianthes heteroclita* | - | JQ589229 | JQ594129 |
| Solanaceae | *Lycianthes multiflora* | - | JQ589232 | JQ594130 |
| Solanaceae | *Lycianthes pauciflora* | - | JQ589253 | JQ594154 |
| Solanaceae | *Lycianthes rantonnei* | - | EF537320 | - |
| Solanaceae | *Lycium afrum* | - | AB036625 | JQ412384 |
| Solanaceae | *Lycium ameghinoi* | - | AB036626 | - |
| Solanaceae | *Lycium americanum* | - | AB036627 | HQ216128 |
| Solanaceae | *Lycium andersonii* | - | AB036628 | AB051024 |
| Solanaceae | *Lycium australe* | - | AB036629 | - |
| Solanaceae | *Lycium barbarum* | - | AB036630 | JF942335 |
| Solanaceae | *Lycium berlandieri var. berlandieri* | - | AB036632 | - |
| Solanaceae | *Lycium carolinianum var. carolinianum* | - | AB036635 | - |
| Solanaceae | *Lycium carolinianum var. quadrifidum* | - | AB036634 | - |
| Solanaceae | *Lycium cestroides* | - | AB036636 | LCU08613 |
| Solanaceae | *Lycium chinense* | - | AB036637 | AB051022 |
| Solanaceae | *Lycium cinereum* | - | AB036623 | EU042185 |
| Solanaceae | *Lycium elongatum* | - | AB036638 | - |
| Solanaceae | *Lycium europaeum* | - | AB036639 | - |
| Solanaceae | *Lycium ferocissimum* | - | AB036640 | AM235152 |
| Solanaceae | *Lycium morongii* | - | AB036641 | - |
| Solanaceae | *Lycium oxycarpum* | - | JX517868 | JX572740 |
| Solanaceae | *Lycium pallidum* | - | AB036642 | AB051025 |
| Solanaceae | *Lycium pilifolium* | - | AB036621 | - |
| Solanaceae | *Lycium prunus-spinosa* | - | AB036620 | - |
| Solanaceae | *Lycium ruthenicum* | - | AB036643 | JF942340 |
| Solanaceae | *Lycium sandwicense* | - | AB036644 | AB051023 |
| Solanaceae | *Lycium schizocalyx* | - | AB036622 | - |
| Solanaceae | *Lycium villosum* | - | AB036624 | - |
| Solanaceae | *Mandragora officinarum* | - | AJ585883 | MOU08614 |
| Solanaceae | *Margaranthus solanaceus* | - | EF438850 | - |
| Convolvulaceae | *Maripa nicaraguensis* | - | JQ587303 | JQ591162 |
| Convolvulaceae | *Merremia aegyptia* | - | JQ587305 | AY100980 |
| Convolvulaceae | *Merremia tuberosa* | - | GU135052 | GU135216 |
| Solanaceae | *Nicandra physalodes* | - | EF438840 | NPU08615 |
| Solanaceae | *Nicotiana acaulis* | - | AB039985 | - |
| Solanaceae | *Nicotiana acuminata* | - | AJ585849 | - |
| Solanaceae | *Nicotiana africana* | - | AJ585881 | - |
| Solanaceae | *Nicotiana alata* | - | AB040000 | - |
| Solanaceae | *Nicotiana amplexicaulis* | - | AB040019 | - |
| Solanaceae | *Nicotiana arentsii* | - | AJ585844 | - |
| Solanaceae | *Nicotiana attenuata* | - | AB040009 | - |
| Solanaceae | *Nicotiana attenuata* | - | AJ585837 | - |
| Solanaceae | *Nicotiana benavidesii* | - | AB039991 | - |
| Solanaceae | *Nicotiana benthamiana* | - | AB040014 | - |
| Solanaceae | *Nicotiana bonariensis* | - | AB039986 | - |
| Solanaceae | *Nicotiana cavicola* | - | AB040016 | - |
| Solanaceae | *Nicotiana clevelandii* | - | AJ585850 | - |
| Solanaceae | *Nicotiana cordifolia* | - | AJ585851 | - |
| Solanaceae | *Nicotiana corymbosa* | - | AJ585852 | - |
| Solanaceae | *Nicotiana debneyi* | - | AB040017 | TOBCPRBCL |
| Solanaceae | *Nicotiana digluta* | - | AJ585853 | - |
| Solanaceae | *Nicotiana exigua* | - | AB040022 | - |
| Solanaceae | *Nicotiana forgetiana* | - | AB040001 | - |
| Solanaceae | *Nicotiana fragrans* | - | AB040023 | - |
| Solanaceae | *Nicotiana glauca* | - | AB039987 | JQ412399 |
| Solanaceae | *Nicotiana glutinosa* | - | AB039995 | - |
| Solanaceae | *Nicotiana goodspeedii* | - | AJ585890 | - |
| Solanaceae | *Nicotiana gossei* | - | AB040018 | - |
| Solanaceae | *Nicotiana kawakamii* | - | AJ585845 | - |
| Solanaceae | *Nicotiana knightiana* | - | AB039989 | - |
| Solanaceae | *Nicotiana langsdorffii* | - | AB039999 | - |
| Solanaceae | *Nicotiana linearis* | - | AB040011 | - |
| Solanaceae | *Nicotiana longiflora* | - | AB040002 | - |
| Solanaceae | *Nicotiana maritima* | - | AJ585884 | - |
| Solanaceae | *Nicotiana megalosiphon* | - | AB040021 | - |
| Solanaceae | *Nicotiana miersii* | - | AB040010 | - |
| Solanaceae | *Nicotiana nesophila* | - | AJ585854 | - |
| Solanaceae | *Nicotiana noctiflora var. albiflora* | - | AB040007 | - |
| Solanaceae | *Nicotiana noctiflora var. noctiflora* | - | AB040006 | GQ248655 |
| Solanaceae | *Nicotiana nudicaulis* | - | AB040013 | - |
| Solanaceae | *Nicotiana occidentalis* | - | AJ585889 | - |
| Solanaceae | *Nicotiana otophora* | - | AB039994 | - |
| Solanaceae | *Nicotiana palmeri* | - | AJ585838 | - |
| Solanaceae | *Nicotiana paniculata* | - | AB039988 | - |
| Solanaceae | *Nicotiana pauciflora* | - | AJ585839 | - |
| Solanaceae | *Nicotiana petunioides* | - | AB040008 | GQ248656 |
| Solanaceae | *Nicotiana plumbaginifolia* | - | AB040003 | - |
| Solanaceae | *Nicotiana quadrivalvis var. bigelovii* | - | AB040012 | - |
| Solanaceae | *Nicotiana raimondii* | - | AJ585840 | - |
| Solanaceae | *Nicotiana repanda* | - | AB040004 | - |
| Solanaceae | *Nicotiana rosulata* | - | AJ585885 | - |
| Solanaceae | *Nicotiana rotundifolia* | - | AJ585886 | - |
| Solanaceae | *Nicotiana rustica* | - | AB039992 | - |
| Solanaceae | *Nicotiana simulans* | - | AB040020 | - |
| Solanaceae | *Nicotiana solanifolia* | - | AB039990 | - |
| Solanaceae | *Nicotiana stocktonii* | - | AB040005 | - |
| Solanaceae | *Nicotiana suaveolens* | - | AJ585842 | - |
| Solanaceae | *Nicotiana sylvestris* | - | AB039998 | - |
| Solanaceae | *Nicotiana tabacum* | - | JN114764 | KC825342 |
| Solanaceae | *Nicotiana thyrsiflora* | - | AJ585888 | - |
| Solanaceae | *Nicotiana tomentosa* | - | AB039993 | - |
| Solanaceae | *Nicotiana tomentosiformis* | - | AJ585847 | - |
| Solanaceae | *Nicotiana trigonophylla* | - | AB039997 | - |
| Solanaceae | *Nicotiana umbratica* | - | AB040015 | - |
| Solanaceae | *Nicotiana undulata* | - | AB039996 | - |
| Solanaceae | *Nicotiana velutina* | - | AJ585887 | - |
| Solanaceae | *Nicotiana wigandioides* | - | AJ585843 | - |
| Solanaceae | *Nierembergia frutescens* | - | EF438884 | - |
| Solanaceae | *Nierembergia veitchii* | - | DQ995350 | - |
| Solanaceae | *Nolana albescens* | - | AB036647 | AB051021 |
| Solanaceae | *Nolana humifusa* | - | EF438984 | - |
| Solanaceae | *Nolana paradoxa* | - | EF438866 | - |
| Solanaceae | *Nolana rostrata* | - | AB036648 | - |
| Solanaceae | *Petunia altiplana* | - | AB262052 | - |
| Solanaceae | *Petunia axillaris* | - | HQ384562 | HQ384915 |
| Solanaceae | *Petunia axillaris subsp. axillaris* | - | AB262053 | - |
| Solanaceae | *Petunia axillaris subsp. parodii* | - | AB262054 | - |
| Solanaceae | *Petunia axillaris subsp. subandina* | - | AB262055 | - |
| Solanaceae | *Petunia bajeensis* | - | AB262056 | - |
| Solanaceae | *Petunia bonjardinensis* | - | AB262057 | - |
| Solanaceae | *Petunia exserta* | - | AB262058 | - |
| Solanaceae | *Petunia guarapuavensis* | - | AB262059 | - |
| Solanaceae | *Petunia integrifolia subsp. depauperata* | - | AB262062 | - |
| Solanaceae | *Petunia integrifolia subsp. inflata* | - | AB262060 | - |
| Solanaceae | *Petunia integrifolia subsp. integrifolia* | - | AB262061 | - |
| Solanaceae | *Petunia interior* | - | AB262063 | - |
| Solanaceae | *Petunia littoralis* | - | AB262064 | - |
| Solanaceae | *Petunia mantiqueirensis* | - | AB262065 | - |
| Solanaceae | *Petunia occidentalis* | - | AB262066 | - |
| Solanaceae | *Petunia reitzii* | - | AB262067 | - |
| Solanaceae | *Petunia riograndensis* | - | AB262068 | - |
| Solanaceae | *Petunia saxicola* | - | AB262069 | - |
| Solanaceae | *Petunia scheideana* | - | AB262070 | - |
| Solanaceae | *Physalis acutifolia* | - | EF438820 | - |
| Solanaceae | *Physalis aequata* | - | EF438883 | - |
| Solanaceae | *Physalis alkekengi* | - | AM503827 | PAU08617 |
| Solanaceae | *Physalis angulata* | - | EF438827 | KC551924 |
| Solanaceae | *Physalis coztomatl* | - | EF438951 | - |
| Solanaceae | *Physalis crassifolia* | - | EF438973 | - |
| Solanaceae | *Physalis curassavica* | - | EF438880 | - |
| Solanaceae | *Physalis divaricata* | - | JX996066 | JX996060 |
| Solanaceae | *Physalis fuscomaculata* | - | EU128754 | - |
| Solanaceae | *Physalis heterophylla* | - | HQ593385 | HQ590207 |
| Solanaceae | *Physalis ignota* | - | JQ589235 | JQ594132 |
| Solanaceae | *Physalis ixocarpa* | - | EF438947 | - |
| Solanaceae | *Physalis lanceifolia* | - | EF438914 | - |
| Solanaceae | *Physalis longifolia var. subglabrata* | - | EU128756 | - |
| Solanaceae | *Physalis mendocina* | - | EF438844 | - |
| Solanaceae | *Physalis mexicana* | - | EF438845 | - |
| Solanaceae | *Physalis minima* | - | EF438846 | - |
| Solanaceae | *Physalis nicandroides* | - | EF438989 | - |
| Solanaceae | *Physalis peruviana* | - | EF438899 | FJ914181 |
| Solanaceae | *Physalis philadelphica* | - | EF438955 | - |
| Solanaceae | *Physalis pruinosa* | - | EF438926 | - |
| Solanaceae | *Physalis pubescens* | - | EF438974 | GQ436616 |
| Solanaceae | *Physalis viscosa* | - | EF438959 | - |
| Solanaceae | *Physochlaina orientalis* | - | EF439009 | HQ216140 |
| Solanaceae | *Physochlaina physaloides* | - | EF439016 | HQ216141 |
| Solanaceae | *Salpichroa origanifolia* | - | EF439048 | HM850331 |
| Solanaceae | *Salpiglossis sinuata* | - | EF439055 | SSU08618 |
| Solanaceae | *Schizanthus grahamii* | - | EF439051 | - |
| Solanaceae | *Schizanthus hookeri* | - | EF439052 | - |
| Solanaceae | *Schizanthus pinnatus* | - | HQ384564 | AY101063 |
| Solanaceae | *Schwenkia americana* | - | JQ589236 | JQ594138 |
| Solanaceae | *Solandra guttata* | - | KC146606 | - |
| Solanaceae | *Solandra maxima* | - | KC146607 | - |
| Solanaceae | *Solanum accrescens* | - | JQ589239 | JQ594139 |
| Solanaceae | *Solanum aculeastrum* | - | JQ024998 | JQ025091 |
| Solanaceae | *Solanum aculeatissimum* | - | JF956480 | JF944443 |
| Solanaceae | *Solanum aethiopicum* | - | KC535801 | KC535808 |
| Solanaceae | *Solanum americanum* | - | JN545023 | JN545017 |
| Solanaceae | *Solanum amotapense* | - | EF439041 | - |
| Solanaceae | *Solanum anguivi* | - | JX511977 | JX511989 |
| Solanaceae | *Solanum aphyodendron* | - | JQ589579 | - |
| Solanaceae | *Solanum arboreum* | - | JQ589240 | JQ594142 |
| Solanaceae | *Solanum asperum* | - | GQ982096 | GQ981876 |
| Solanaceae | *Solanum asymmetriphyllum* | - | EU983570 | - |
| Solanaceae | *Solanum atropurpureum* | - | EF439040 | - |
| Solanaceae | *Solanum aturense* | - | JQ589576 | JQ594143 |
| Solanaceae | *Solanum betaceum* | - | EF438983 | - |
| Solanaceae | *Solanum bulbocastanum* | - | EF439049 | - |
| Solanaceae | *Solanum candidum* | - | JQ589243 | JQ594148 |
| Solanaceae | *Solanum carduiforme* | - | EU983558 | - |
| Solanaceae | *Solanum catombelense* | - | JF270941 | JF265599 |
| Solanaceae | *Solanum chenopodioides* | - | JN545025 | HM850361 |
| Solanaceae | *Solanum chippendalei* | - | EU983562 | - |
| Solanaceae | *Solanum chrysotrichum* | - | HM851099 | HM850362 |
| Solanaceae | *Solanum cinereum* | - | EU983575 | - |
| Solanaceae | *Solanum circinatum* | - | GQ982097 | GQ981877 |
| Solanaceae | *Solanum clarkiae* | - | EU983564 | - |
| Solanaceae | *Solanum clavatum* | - | KC535796 | KC535804 |
| Solanaceae | *Solanum cordovense* | - | JQ589248 | JQ594149 |
| Solanaceae | *Solanum dasyphyllum* | - | EF439020 | - |
| Solanaceae | *Solanum dioicum* | - | JN098479 | - |
| Solanaceae | *Solanum diphyllum* | - | GU134970 | GU135130 |
| Solanaceae | *Solanum diversiflorum* | - | EU983563 | - |
| Solanaceae | *Solanum dulcamara* | - | FN668838 | EU677012 |
| Solanaceae | *Solanum elaeagnifolium* | - | EU983576 | - |
| Solanaceae | *Solanum ferocissimum* | - | EF439022 | - |
| Solanaceae | *Solanum furfuraceum* | - | EF439023 | - |
| Solanaceae | *Solanum giganteum* | - | JX517374 | JX572986 |
| Solanaceae | *Solanum hayesii* | - | GQ982098 | JQ594158 |
| Solanaceae | *Solanum hazenii* | - | JQ589257 | JQ594159 |
| Solanaceae | *Solanum heteropodium* | - | EU983567 | - |
| Solanaceae | *Solanum hirsutissimum* | - | EF439025 | - |
| Solanaceae | *Solanum hirtum* | - | EF439026 | - |
| Solanaceae | *Solanum hispidum* | - | EF439027 | - |
| Solanaceae | *Solanum hoplopetalum* | - | EU983571 | - |
| Solanaceae | *Solanum humboldti* | - | EF439075 | - |
| Solanaceae | *Solanum jamaicense* | - | GU135067 | GU135229 |
| Solanaceae | *Solanum jamesii* | - | EF439047 | - |
| Solanaceae | *Solanum kurzii* | - | KC535800 | KC535807 |
| Solanaceae | *Solanum laciniatum* | - | EF439043 | GQ436613 |
| Solanaceae | *Solanum lasiophyllum* | - | EF439029 | - |
| Solanaceae | *Solanum laxum* | - | HM851101 | HM850364 |
| Solanaceae | *Solanum leopoldense* | - | EU983560 | - |
| Solanaceae | *Solanum lepidotum* | - | GQ982099 | GQ981879 |
| Solanaceae | *Solanum lichtensteinii* | - | JF270942 | JF265600 |
| Solanaceae | *Solanum linnaeanum* | - | HM851102 | HM850365 |
| Solanaceae | *Solanum lycopersicum* | - | FN668840 | HF572813 |
| Solanaceae | *Solanum macrocarpon* | - | EF439056 | - |
| Solanaceae | *Solanum manaense* | - | EF439030 | - |
| Solanaceae | *Solanum marginatum* | - | HM851103 | - |
| Solanaceae | *Solanum mauritianum* | - | HM851104 | HM850366 |
| Solanaceae | *Solanum melanospermum* | - | EU983565 | - |
| Solanaceae | *Solanum microdontum* | - | EF439063 | - |
| Solanaceae | *Solanum nigrum* | - | EU749407 | HM850367 |
| Solanaceae | *Solanum oedipus* | - | EU983566 | - |
| Solanaceae | *Solanum oplocense* | - | EF439064 | - |
| Solanaceae | *Solanum panduriforme* | - | EU214285 | EU213514 |
| Solanaceae | *Solanum petraeum* | - | EU983555 | - |
| Solanaceae | *Solanum physalifolium* | - | HQ235336 | HQ644072 |
| Solanaceae | *Solanum pinnatisectum* | - | EF439065 | - |
| Solanaceae | *Solanum pittosporifolium* | - | JF956499 | JF944460 |
| Solanaceae | *Solanum pseudocapsicum* | - | FN668837 | HM850368 |
| Solanaceae | *Solanum ptychanthum* | - | HQ235335 | HQ235620 |
| Solanaceae | *Solanum pubescens* | - | JX511979 | JX511991 |
| Solanaceae | *Solanum racemosum* | - | EF439033 | - |
| Solanaceae | *Solanum ramonense* | - | JQ589569 | JQ594547 |
| Solanaceae | *Solanum renschii* | - | EF439034 | - |
| Solanaceae | *Solanum retroflexum* | - | JN545026 | JN545020 |
| Solanaceae | *Solanum rovirosanum* | - | JQ589264 | JQ594168 |
| Solanaceae | *Solanum rudepannum* | - | JQ589265 | JQ594170 |
| Solanaceae | *Solanum rugosum* | - | JQ589268 | JQ594175 |
| Solanaceae | *Solanum scabrum* | - | JN545028 | JN545022 |
| Solanaceae | *Solanum schlechtendalianum* | - | JQ589270 | JQ594178 |
| Solanaceae | *Solanum sejunctum* | - | EU983568 | - |
| Solanaceae | *Solanum sisymbriifolium* | - | KC535799 | KC535805 |
| Solanaceae | *Solanum spirale* | - | JF956501 | JF944463 |
| Solanaceae | *Solanum stupefactum* | - | EU983572 | - |
| Solanaceae | *Solanum tampicense* | - | GU135074 | GU135238 |
| Solanaceae | *Solanum tequilense* | - | EF439035 | - |
| Solanaceae | *Solanum torvum* | - | GU135088 | JX511993 |
| Solanaceae | *Solanum tridynamum* | - | EF439036 | - |
| Solanaceae | *Solanum trilobatum* | - | JX511982 | JX511994 |
| Solanaceae | *Solanum trisectum* | - | EF438842 | - |
| Solanaceae | *Solanum tuberosum* | - | FN668841 | HF572814 |
| Solanaceae | *Solanum tudununggae* | - | EU983552 | - |
| Solanaceae | *Solanum vespertilio* | - | EF439037 | - |
| Solanaceae | *Solanum viarum* | - | GU135073 | GU135203 |
| Solanaceae | *Solanum villosum subsp. alatum* | - | HE967494 | HE963683 |
| Solanaceae | *Solanum virginianum* | - | JX511980 | JX996057 |
| Solanaceae | *Solanum wrightii* | - | EF439038 | JX856319 |
| Solanaceae | *Symonanthus aromaticus* | - | AJ585878 | - |
| Solanaceae | *Symonanthus bancroftii* | - | AJ585879 | - |
| Solanaceae | *Tubocapsicum anomalum* | - | EF439073 | - |
| Solanaceae | *Vassobia breviflora* | - | EF439077 | - |
| Solanaceae | *Vassobia fasciculata* | - | EF439079 | - |
| Solanaceae | *Vestia lycioides* | - | EF438822 | - |
| Solanaceae | *Withania coagulans* | - | KC291707 | DQ368402 |
| Solanaceae | *Withania frutescens* | - | EF438824 | DQ368401 |
| Solanaceae | *Withania somnifera* | - | JX996061 | DQ353868 |
| Solanaceae | *Witheringia coccoloboides* | - | EF438981 | - |
| Solanaceae | *Witheringia correana* | - | JQ589584 | JQ594550 |
| Solanaceae | *Witheringia solanacea* | - | JQ589277 | JQ594188 |
